# Reports of Medical Society Proceedings

**Published:** 1881-03

**Authors:** 


### CORRESPONDENCE.

### REPORTS OF MEDICAL SOCIETY PROCEEDINGS.

MR. EDITOR.—When I read your objections to publishing the proceedings of certain Medical Societies, in the INDEPENDENT PRACTITIONER, some time ago, I thought you bore down rather heavily upon some speaking members then in my mind's eye; and I determined to see some of the published accounts of those in our midst. Now, I am free to confess that you did not over-state the utter valuelessness of some of them. Indeed, I think it a useless consumption of journal space, and in no-wise calculated to inspire confidence in medicine as a science, to read such statements as I shall introduce presently; certainly the practitioner who reads them will not find anything either to aid him in his cases, or to increase his confidence in, or his respect for, the medical profession in our midst; i. e. if the following extracts from a report of *one* of our local societies, in a Philadelphia journal, are reliable! viz: "*Diphtheria*," Dr. P., "Sulphate Copper was used liberally, but it did not seem to answer the purpose; nitrate of silver, (ʒ i aq. ℥ i) was used instead. As prophylactics, the best of food and brandy were used freely. I had fifteen cases and *not one* of them died." Dr. E. "prophylaxis may be of some use," "I have *ceased to dread* diphtheria since adopting my present mode of treatment. *Formerly* I used nitrate of silver, but the struggling of the child seemed to do as much harm as the application did good. *I use*—℞. Quinia, gr. viii, tint. ferri chlor, ʒ i, syr. simp. ℥ iv. M. Sig.—Teaspoonful every hour, day and night. Since I have adopted *this* method, the results have been *astonishing*. I have used it four years, and have only lost *three cases*." Dr. A., "The treatment of diphtheria is the opprobium of medicine; under two years it is an extremely fatal disease. Those cases associated with croup, always die." Dr. M., "During the last two years *I* have used *no* iron, quinia, *nor* local applications in this disease. *I use* ice, food and lemon juice freely, and have had *better success* than formerly." Surely this society could not have approved of the publication of such absurdities and contradictions as the foregoing. In the interests of science and humanity, it were well their portals were securely guarded against the promulgation of such strangely contradictory statements and assertions. I am fully convinced now, that you are right in excluding such proceedings from your journal.

Yours gratefully, SYNDROME.

